# The Microbeam Insert at the White Beam Beamline P61A at the Synchrotron PETRA III/DESY: A New Tool for High Dose Rate Irradiation Research

**DOI:** 10.3390/cancers14205137

**Published:** 2022-10-20

**Authors:** Elisabeth Schültke, Stefan Fiedler, Catharina Mewes, Elisabetta Gargioni, Johannes Klingenberg, Guilherme Abreu Faria, Michael Lerch, Marco Petasecca, Franziska Prehn, Marie Wegner, Marten Scholz, Felix Jaekel, Guido Hildebrandt

**Affiliations:** 1 Department of Radiooncology, Rostock University Medical Center, 18059 Rostock, Germany; 2 European Molecular Biology Laboratory (EMBL), Hamburg Outstation, 22607 Hamburg, Germany; 3 Department of Radiotherapy and Radio-Oncology, University Medical Center Hamburg-Eppendorf, 20246 Hamburg, Germany; 4 Helmholtz-Zentrum HEREON, 21502 Hamburg, Germany; 5 Centre of Medical Radiation Physics, University of Wollongong, Wollongong 2522, Australia; 6 Institute of Product Development and Mechanical Engineering Design, Hamburg University of Technology, 21073 Hamburg, Germany

**Keywords:** high dose rate radiotherapy, FLASH radiotherapy, microbeam radiotherapy (MRT), synchrotron

## Abstract

**Simple Summary:**

The excellent preservation of normal tissue function after high dose rate radiotherapy has been shown in pre-clinical studies. Normal tissue in the tumor environment is well preserved even after target doses of several hundred Gy while reliably destroying the tumor cells. These results have triggered the establishment of appropriate research structures at the synchrotron PETRA III on the DESY campus in Hamburg, Germany. Dose rates of hundreds of Gy/s can be achieved, compared to 6–20 Gy/min in clinical radiotherapy. We describe the design, development, key parameters, and first use of a mobile insert for high dose rate radiotherapy research, a new research instrument at P61A, the first polychromatic beamline of PETRA III. The data obtained at the end station P61A will support the international interdisciplinary effort to improve radiotherapy concepts for patients with malignant tumors that are considered radioresistant with the currently established clinical radiotherapy techniques.

**Abstract:**

High dose rate radiotherapies such as FLASH and microbeam radiotherapy (MRT) both have developed to the stage of first veterinary studies within the last decade. With the development of a new research tool for high dose rate radiotherapy at the end station P61A of the synchrotron beamline P61 on the DESY campus in Hamburg, we increased the research capacity in this field to speed up the translation of the radiotherapy techniques which are still experimental, from bench to bedside. At P61, dose rates of several hundred Gy/s can be delivered. Compared to dedicated biomedical beamlines, the beam width available for MRT experiments is a very restrictive factor. We developed two model systems specifically to suit these specific technical parameters and tested them in a first set of experiments.

## 1. Introduction

The advent of medical linear accelerators (LINACS) enabled the production of X-rays with qualities in the MV range. With the associated increase of penetration depth and reduced energy absorption in the tissue between body surface and target, the risk of adverse effects decreased. In combination with modern irradiation techniques like image guidance during dose delivery and integrated dose escalation, higher total target doses could be administered for treating deep-seated tumors, which subsequently resulted in better tumor control [1,2,3,4,5]. However, despite these advances in modern clinical radiotherapy, tumor entities like glioblastoma multiforme, sarcoma, and melanoma are still considered highly radioresistant [6,7]. In other words, due to dose limitations dictated by the threshold of normal tissue tolerance, the X-ray doses that can be safely administered with clinically established radiotherapy techniques are not high enough to ensure a favorable prognosis. The introduction of high dose rate radiotherapy could help to improve tumor control in these cases by enabling the delivery of higher target doses while preserving normal tissue function. Data obtained from small animal studies of glioblastoma and melanoma support this hypothesis [8,9,10].

It has been reported that the administration of high single fraction doses at dose rates of ≥40 Gy results in extremely good protection of normal tissue morphology and function [11,12,13]. The dose rates in clinically established radiotherapy techniques, in comparison, are approx. 6–20 Gy/min. FLASH radiotherapy, using this tissue protective effect, has developed within only one decade from a preclinical experimental concept to the stage of veterinary trials [14,15,16]. One human patient has already been treated successfully [17]. 

The second high dose rate radiotherapy technique which has recently matured from work in small animal models of malignant disease to the stage of veterinary trials is microbeam radiotherapy (MRT). Different to FLASH radiotherapy, which is a broad beam or seamless beam irradiation technique, MRT is characterized by spatial fractionation at the micrometer range. The irradiation target is covered by an array of quasi-parallel microbeams, which creates a repetitive pattern of high dose (peak dose) zones and low dose (valley dose) zones in the tissue. It has been shown in small animal models that MRT, alone or in combination with a course of conventional radiotherapy, can control malignant tumors much better than conventional irradiation alone [18,19,20]. The first veterinary patient with a malignant brain tumor was successfully treated with MRT in September 2021 [21]. 

Internationally, there are currently two biomedical beamlines dedicated to the development of microbeam radiotherapy: ID17 at the Europeans Synchrotron Radiation Facility (ESRF) in France and the Imaging and Biomedical Beamline (IMBL) at the Australian Synchrotron in Melbourne [22,23,24]. Microbeam studies have also been conducted at Spring 8 [25,26,27] and the feasibility to conduct MRT at the biomedical beamline SYRMEP at the Elettra Sincrotrone Trieste in Italy has been demonstrated [28]. 

In order to further increase the research capacity for high dose rate radiotherapy techniques and to speed up the development towards human clinical trials, we explored the opportunity to conduct high dose rate radiotherapy research at the synchrotron PETRA III. In August 2016, we conducted a first MRT feasibility study at the monochromatic beamline P07. Based on the results of that experiment and subsequent studies at the monochromatic beamline P21.2 [29], we decided to develop a dedicated setup for biomedical high dose rate irradiation studies at the new white beam beamline P61A which was, at that time, designed and constructed under the leadership of the Helmholtz Centre Geesthacht (now HEREON). The safety tests for this new beamline were passed in November 2020. In April 2021, we carried out the first external user group experiment at the beamline P61A. This is the first report on beamline parameters important for FLASH and microbeam irradiation studies. Examples for biomedical studies, adapted to the specific technical parameters of this beamline, are shown. This beamline can now be used by the biomedical user community.

## 2. Materials and Methods

### 2.1. Beamline Design

**Figure 1:**
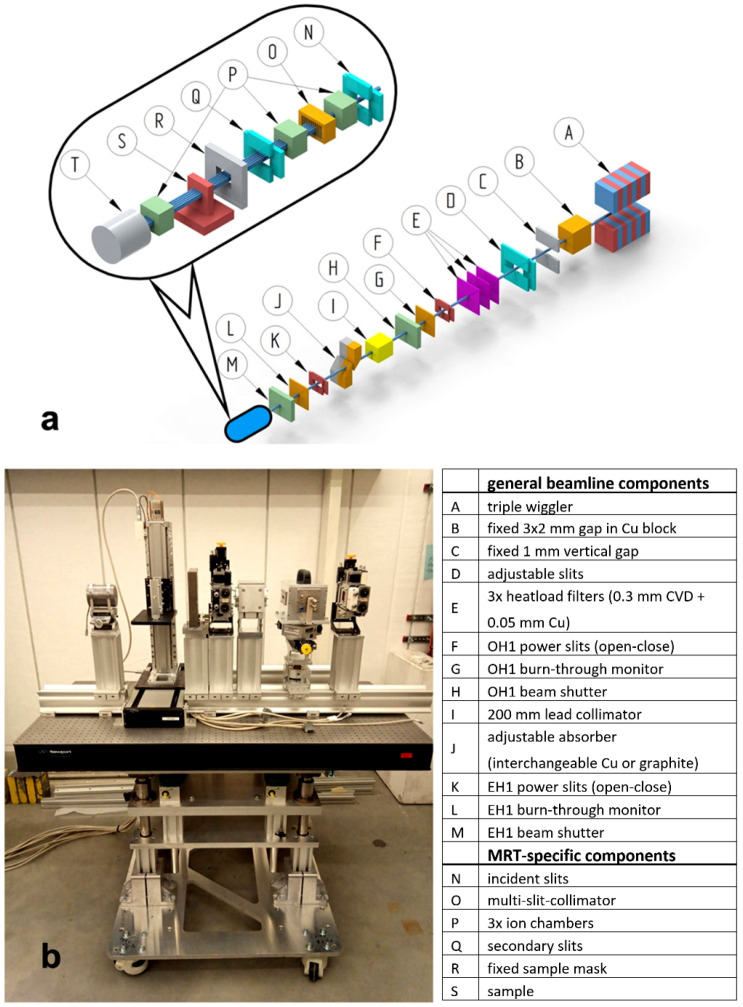
Design of Beamline P61A. Schematic of the beamline (**a**), the parts for the mobile insert for biomedical work are depicted as N-T and the insert that is located at a distance of 108 m from the last wiggler source is shown in the photograph (**b**).

The beamline P61 is the first polychromatic beamline at the synchrotron PETRA III on the DESY campus in Hamburg. The frontend components have been described in detail already [30]. A schematic of the beamline components is shown in Figure 1a. 

Briefly, the X-ray beam available from the PETRA III storage ring is produced by a series of ten damping wigglers (element A in Figure 1a). A series of fixed size apertures and slits limit the available beam size in order to reduce the integrated power load of the wigglers (elements B, C, D, F in Figure 1a). To prevent overheating of the beamline components, three heat-load filters are available (element E in Figure 1a). In addition, adjustable thickness Cu attenuators can be used to shape the energy spectrum (element J in Figure 1a). The components of the mobile insert for biomedical work are located at 108 m distance from the last wiggler (elements N-T in Figure 1a and photograph Figure 1b).

### 2.2. Beam Parameters and Spectrum

The wiggler section that provides the beam to P61 was originally installed to reduce the emittance of the electron beam in the storage ring [31]. Each of the 10 wigglers is 4 m long with a magnetic period of 200 mm, peak magnetic field of 1.52 T, and deflection parameter of 28.4. The ten wigglers are positioned in line in the north straight section of the PETRA III ring. Due to the 6 GeV energy of the electron beam, they deliver an outstanding X-ray photon flux in a broad energy spectrum, reaching up to several hundred keV. 

For MRT irradiations, a flat top beam field is desirable which is achieved at P61 due to the large distance from source to experiment and the high collimation of the small radiation field. The divergence of the beam emitted by each wiggler is 4 × 0.1 mrad^2^. The divergence at the sample position is ultimately defined by the apertures and the alignment of the electron trajectory through the ten wigglers. When the trajectory is perfectly aligned and the apertures are at their maximum positions, the divergence on the sample position is around 20 × 60 μrad^2^. 

The large flux produced by the insertion devices at P61A has, as a consequence, large dose rates and high-power density of the X-ray beam incident on the sample. An integrated power density of 20 W/mm^2^ at the sample position in the experimental hutch is available in the central cone of the beam [30]. Several components are installed along the beamline to handle this power load and protect downstream instruments. These include three 0.3 mm thick CVD diamond filters coated in Cu (0.05 mm). The use of at least one of them is mandatory for normal beamline operation. In addition, several fixed gap apertures limit the beam size in the P61A experimental hutch to approximately 2.8 × 1.8 mm^2^ and reduce the power. Furthermore, a wedge-shaped variable thickness attenuator is installed in the optics hutch to adjust the beam spectrum and intensity depending on experimental requirements. The absorber material is interchangeable between Cu and graphite, and the thickness inserted into the beam can be varied continuously between 0 and 100 mm. As the specific settings of filters modify the spectrum available for an experiment, the energy spectrum for different parameters was calculated. 

**Figure 2:**
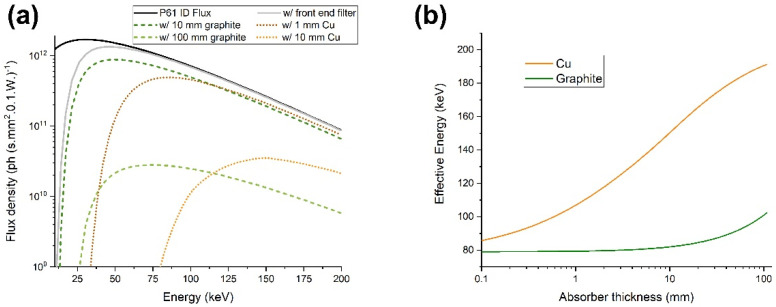
(**a**) Simulated energy spectrum at sample position, at Beamline P61A. Values considering a frontend filter and different thicknesses of absorber materials are also shown. (**b**) Effective energy (weighted average of attenuated spectrum) as a function of absorber thickness, calculated for Cu and graphite.

Figure 2 shows the calculated flux density at the P61A beamline’s experimental hutch position as a function of energy for different filter settings. Flux was determined by combining the independently calculated flux for each wiggler unit considering their relative distances to the experiment hutch. Each wiggler is positioned at roughly 6 m from the next, with the last one being at 100 m from the experimental hutch. The incident flux was calculated for the PETRA III and P61A insertion device parameters with 100 mA. This corresponds to the beam current during the experiments reported in the experimental section. Flux was calculated using the pre-built wiggler model on the Spectra software package [32], which is based on analytical equations describing synchrotron radiation. The flux attenuated by the filter and the variable thickness absorber was calculated using X-ray mass attenuation coefficients provided by NIST [33]. 

### 2.3. Experimental Setup for MRT

Experiments are carried out in heavily shielded experimental hutches (EH) following the concept already applied for the optical hutches of the other PETRA III beamlines [34]. Barite-infused, up to 500 mm thick concrete walls and doors with up to 25 mm lead shielding contain all the high energy scattered radiation inside the EH. 

The frontend of the P61 beamline serves two end stations. End station P61B, containing a large-volume multi-anvil press, was described in detail by Farla [30]. The end station P61A is operated by the Helmholtz Centre HEREON (formerly Helmholtz Centre Geesthacht) and has been designed for material sciences research. Nonetheless, one main focus of its design was versatility in the experiments it can accommodate. As part of this effort, a 3 m long section is defined in the experimental hutch where user supplied equipment can be assembled, with full possibility of incorporating motor control and data collection within the beamline control framework. The challenges for the experimental setup include time management and reproducibility of the setup. Experimental time is awarded in a highly competitive process and a decrease in required setup time leaves more time for the actual study to be conducted. A setup in which all the technical key components are fixed relative to each other, and which is referenced to the absolute beam position, helps to satisfy these requirements. For the high dose rate radiotherapy work at end station P61A, we chose a roll-on/roll-off solution which is described here in detail as a reference for future experiments that will be carried out with this setup. 

The setup for high dose rate studies is assembled on a motorized height-adjustable table. Set on wheels, it can be easily moved in and out of the hutch. Extendable heavy-load feet assure the stable positioning once the setup is aligned inside the hutch. A large honeycomb optical breadboard with a size of 1.50 × 0.75 m^2^ serves as a tabletop. All further components are mounted either directly onto the breadboard or on a system of aluminum profile rails and carriers. All motorized elements of the set-up are integrated into the general beamline motion control.

The further description of the individual components in this setup is divided into three sections in the beam direction downstream from the source: (1) the pre-sample section, where the incident broad beam can be monitored and shaped into microbeams; (2) the sample environment, where a range of dosimeters can be toggled into the beam or the sample can be irradiated; (3) the post-sample section.

(1) In detail, the pre-sample section consists of the following components, described in the beam direction:
  - Incident and secondary slits: The motorized slits (IB-C30-AIR, JJ X-ray A/S, Hørsholm, Denmark) are composed of four 10 mm thick, polished tungsten carbide blades that are slightly pitched for total reflection rejection. The slit system is flushed with gaseous nitrogen to slow beam-induced oxidation processes on the blades. The whole slit assembly can be adjusted in height via a vertically mounted linear stage. The first slits are used to further shape the beam coming from the P61A optics hutch and to remove possible uncollimated and scattered radiation. The purpose of the secondary slits is to remove scattered radiation generated by the multislit collimator. In addition, they can be used for beam characterisation by scanning the slits, when closed to a few micrometres.
  - Multi Slit Collimator (MSC): the fixed-spacing multislit collimator (UNT, Morbier, France) collimates the incident broad beam into an array of quasi-parallel, 50 µm wide microbeams spaced at a 400 µm center-to-center distance [35]. The slits are cut into an 8 mm thick tungsten block. The water cooled MSC is inserted into an inhouse built, nitrogen flushed chamber. It is mounted on a stage and thus can be rotated around its vertical axis as well as moved vertically and horizontally. The rotation stage is used to align the yaw direction of the collimator with the microslit lamellae precisely parallel to the beam direction and with the lateral translation symmetrically to the center of the beam so that uniform and well collimated microbeams can be obtained.
  - Ion chambers: The ion chambers were designed and manufactured by DESY. They are part of the P61A standard equipment used for intensity monitoring of the high energy beam and integrated into the beamline data acquisition system (40 mm electrode length, 6 mm electrode distance).
  - Fixed sample mask: Because of the considerable radiation background produced along the in-air beam path, a large anti-scatter shielding block, made of a massive lead-iron plate with an aperture, is installed in front of the sample stage.
(2) Different sample setups can be placed on a small bread board table and positioned and translated vertically through the beam by a linear stage with a large stroke (Aerotech ECO115SL, Aerotech, Inc., Pittsburg, PA, USA). The stage has a load capacity of up to 5 kg and can be driven with up to 100 mm/s. The wide adjustability of the speed allows the variation of the dose by approximately two orders of magnitude when the sample is moved during an exposure vertically through the fixed synchrotron beam. The vertical stage itself is mounted onto a large stroke horizontal translation. In this manner the sample setup can be positioned within a rectangular range of 320 × 480 mm^2^ (horizontal × vertical).
(3) The post-sample section contains another ion chamber for transmission measurements. Additionally, a scintillator-based X-ray camera for coarse sample imaging and beam alignment can be moved into the beam path. The CMOS camera (UI-5880CP-C-HQ Rev.2, IDS Imaging Development Systems GmbH, Obersulm, Germany) has a 5 times magnified optic and therefore an effective pixel size of 2.5 µm. A massive lead beam stop is installed at the end of the beam path.

### 2.4. Dosimetry: Measurements, Recordings, and Monte Carlo Simulation

Dose measurements were carried out using a commercially available probe, a microDiamond detector with a sensitive volume of 1.1 mm in radius and 1 µm in thickness (PTW microDiamond TM 60019, Freiburg, Germany), a silicon strip detector (EPI-50) with a width of 10 µm fabricated on an epitaxial substrate of 50 µm in thickness, and self-developing radiochromic film of the Gafchromic™ HD-V2 type (Ashland, Bridgewater, MA, USA).

Dose rates of several hundred of Gy/s can be adjusted by varying the thickness of the Cu filters. Using a 20 mm Cu absorber, a dose rate of 1 kGy/s is measured with the microDiamond chamber. Higher dose rates are possible, but the risk of radiation damages to the equipment increases and the saturation threshold of the dosimeter is reached. Integrated doses measured with the microDiamond chamber are verified with the other cross-calibrated dosimeters. 

**Figure 3:**
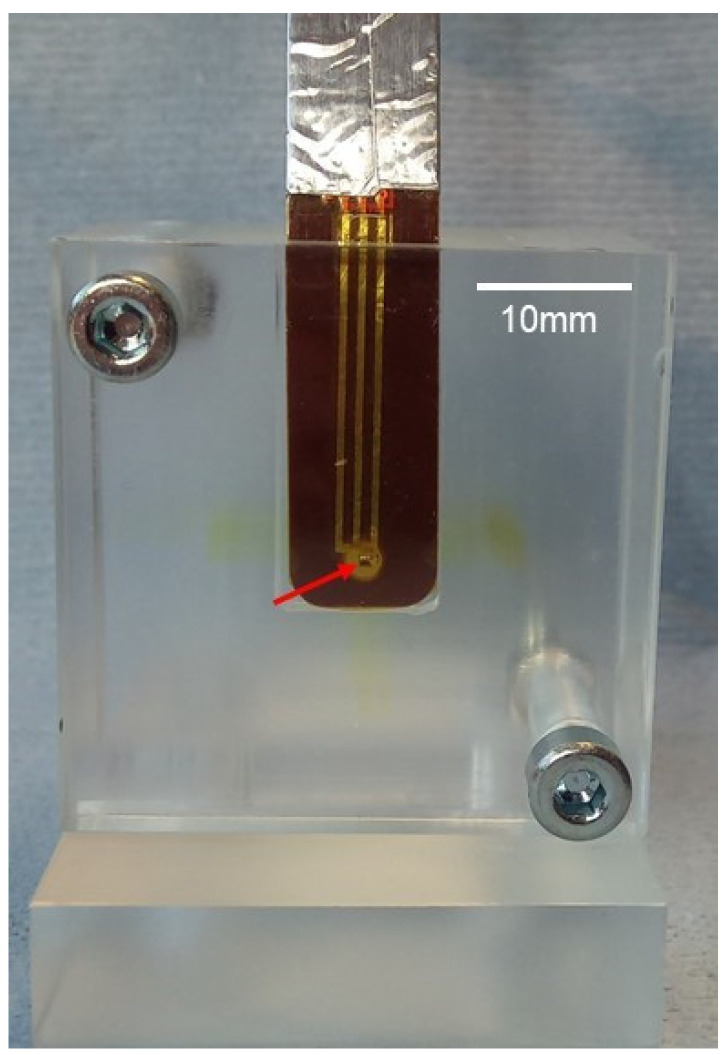
EPI-50 silicon strip detector embedded in a PMMA phantom in face-on view. The arrow indicates the approximate position of the sensitive area. The brownish staining in the PMMA was caused by a short exposure of the phantom to the X-ray beam.

The EPI-50 silicon strip detector is embedded in a PMMA phantom (Figure 3). The sensitive volume of the EPI-50 detector is exposed to the beam with the orientation that maximises its spatial resolution, corresponding to an end-on edge-on positioning in respect to the microbeams. Consequently, the width and the intensity of the microbeams and the ratio between peak dose and valley dose are determined with this system. The detector is read out by the X-Tream dosimetry system (CMRP, University of Wollongong, Wollongong, Australia), a fast, high dynamic range, single channel electrometer. The X-Tream system has been developed specifically for dosimetry in small volumes and allows real-time read-out and can achieve a spatial dose resolution of approximately 6 µm [36]. X-Tream is composed of a transimpedance pre-amplifier connected through a differential analogue interface to a fast (1 MHz) digital converter mastered by a field programmable gate array (FPGA). The FPGA manages also the USB2.0 communication with a host computer which collects the data by a custom design graphical interface. 

Gafchromic™ radiochromic film was used to verify the microbeam geometry. The spatial dose resolution for HD-V2 Gafchromic™ film is approx. 5 µm and the dynamic dose range is 10–1000 Gy, which makes it well suited for recording of microbeams.

As part of the dosimetric characterization, radiation transport simulations were carried out using the Monte Carlo code Geant4 (Version 10.5.1) [37]. For the purpose of this study, we assumed that the photon spectrum corresponds to the one simulated by Farla et al. [30]. In addition, we simulated a non-divergent beam with a rectangular shape of (2.4 × 1.2) mm^2^, as obtained from beam-profile measurements. We did not include any beamline components and for simulations with the MSC, we considered tungsten carbide blocks for the production of microbeams. These blocks are 350 μm wide, 8 mm thick, have a height of 3 mm, and are separated by 400 μm (center-to-center spacing). For the Monte Carlo simulations, ≥10^10^ photons were used to achieve a reasonable relative standard uncertainty (<4%) depending on the chosen thickness of the Cu absorbers.

### 2.5. Cell Culture for Use in the Modified Alderson Phantom

Commercially available human malignant T98G brain tumor cells (ATCC, Manassas, VA, USA) were grown in DMEM (1×) + GlutaMax™-I growth medium with 4.5 g/L D-glucose and pyruvate (catalogue number 31966-021, gibco life technologies) + supplemented by 1% fetal bovine serum (FBS Superior, Sigma Aldrich, S0615, St. Louis, MO, USA) and 10% penicillin/streptomycin (PenStrep, Sigma Aldrich, P4333, St. Louis, MO, USA) in a standard incubator. They were harvested after aspirating the growth medium and incubating with Hanks’ balanced salt solution (HBSS 1×, catalogue number 14175-137, gibco life technologies) in a standard incubator for approx. 20 min, centrifuged for 5 min at 250 rpm and resuspended in 0.4% agar dissolved in growth medium.

Tubes to contain the 3D cell cultures were printed in an additive manufacturing process (BCN3D Sigma R19, BCN3D Technologies, Gavà, Spain) from biocompatible material (Extrudr GreenTEC Pro, Extrudr FD3D GmbH, Lauterach, Austria). To allow access to the growth medium, in which the tubes are stored, holes of 1–2 mm diameter perforate the tube walls at regular intervals. 

To fill the tubes, they were placed vertically with one open end in an approx. 2 mm high base of 2% agar (catalogue number 4508.1, Carl Roth GmbH, Karlsruhe, Germany), after the holes in the lateral tube wall were covered thinly with 2% agar solution. The tubes were then filled with the cell-containing agar solution. Cell growth inside the tubes was documented under the microscope three times per week.

## 3. Results

### 3.1. Characterization of Microbeams at Beamline P61A

**Figure 4:**
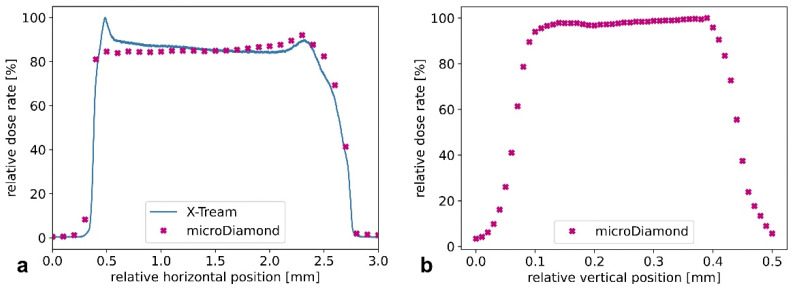
Characterizing the field size and shape at the white-beam beamline with different dosimeters. Horizontal (**a**) and vertical (**b**) profiles, measured around the beam central axis. These data, obtained with the microDiamond (points) and the X-Tream dosimetry system (line), are used as input properties in the Monte Carlo simulation. The error bars are as large as the symbol representing the experimental values.

The lateral and longitudinal field profiles of the broad beam show that it is possible to uniformly irradiate samples with lateral dimensions of approximately 2.4 mm (Figure 4). Also, a vertical scanning of long samples allows for a uniform dose delivery, since the photon flux is homogeneous also in this direction. Based on these measurements, we implemented the corresponding field size in the Monte Carlo simulations.

**Figure 5:**
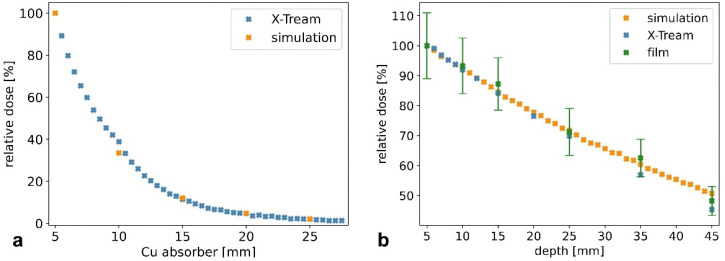
Characterizing the dosimetry properties of the white-beam beamline with different dosimeters and Monte Carlo simulations. Relative dose rate for increasing Cu thickness (**a**). Depth-dose curve in PMMA for a Cu thickness of 10 mm (**b**). The error bars for the measurements with the X-Tream system are as large as the symbols representing the experimental values.

**Figure 6:**
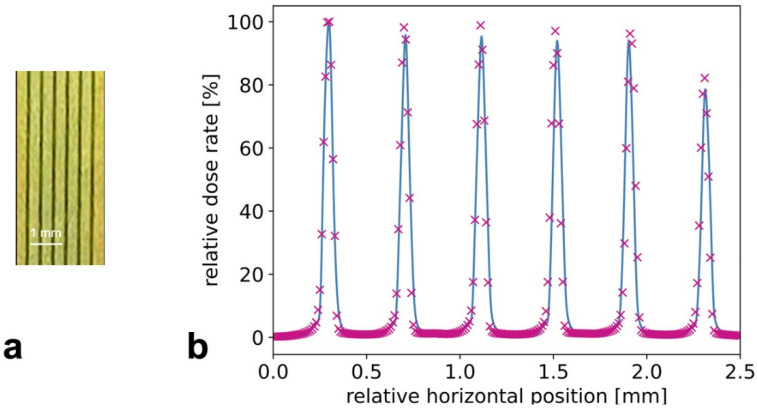
Microbeams obtained at the beamline P61A. Recorded on an HD-V2 Gafchromic™ film (**a**) and relative dose rate measured with the X-Tream (blue) and PTW microDiamond (magenta) (**b**).

The analysis of the dose-rate dependence for several Cu filters is summarized in Figure 5. We achieved a reduction of 67% with respect to the initial beam filtration of 5 mm by using an additional Cu thickness of 5 mm. This experimental result was confirmed by having a very good agreement with the Monte Carlo simulations (see Figure 6). The simulated incident X-ray spectrum is therefore suitable for these preliminary studies. This is confirmed by the good agreement between measured and simulated depth-dose curves in PMMA, as shown exemplarily in Figure 6 for a Cu filtration of 10 mm.

First measurements with the MSC, reported in Figure 6, show that a PVDR of about 150 can be achieved at the phantom surface, while this value would be reduced to about 130 at a depth of 20 mm.

### 3.2. Models Developed for Biomedical Work at Beamline P61A

The challenge for biomedical work at beamline P61A is the narrowness of the incident beam typical for the PETRA III beamlines. Nevertheless, to assure biomedical work of high scientific quality, we designed and developed two model systems specifically suited to the technical conditions in the P61A environment. This will enable independent biomedical experiments at P61A as well as complementary experiments at a beamline with a larger incident beam width or at clinical irradiation facilities.

**Figure 7:**
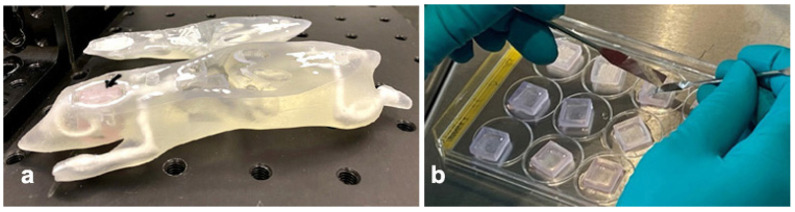
Mouse phantom generated in an additive manufacturing process (3D printing) with tissue equivalent materials, with 3D bioprint generated from primary brain tumor cell inserted (**a**). The black arrow points at the bioprint. Well plate with 3D bioprints in cases (**b**).

With a reduction of the number of animal studies in mind, a mouse phantom was produced in an additive manufacturing process from tissue-equivalent materials [38]. Specifically for work at beamline P61A, a 6 mm × 13 mm wide and 13 mm deep vertical slit was created to insert an approx. 5 × 10 mm sample generated in a 3D bioprinting technique from malignant tumor cells. The bioprinting technique has been previously described [29]. 3D bioprinted material can be reproducibly custom shaped during the printing process, to fit the requirements of each individual experiment. In order to avoid contamination of the phantom or the beamline with biological material, a re-usable case was printed using a 3D printer (Form 2, Formlabs, Somerville, MA, USA) from biocompatible material (BioMed Clear Resin, Formlabs, Somerville, MA, USA). At the cell culture work bench under sterile conditions, the bioprinted material is placed into this case, which in turn is inserted into the mouse phantom (Figure 7). 

The second model uses the commercially available Alderson radiation therapy phantom frequently used for clinical dosimetry, produced from tissue-equivalent material and conforming to ICRU-44 standards. Choosing the slice number 4 of this phantom, a coronary section at the level of the pituitary gland, the 2.5 cm long blank pins were partially replaced by tubes of equal size, containing 3D cell cultures grown in an agar matrix. A coordinate system was overlayed to assure that every single 3D sample could be assigned an identifying position for later analysis. 

**Figure 8:**
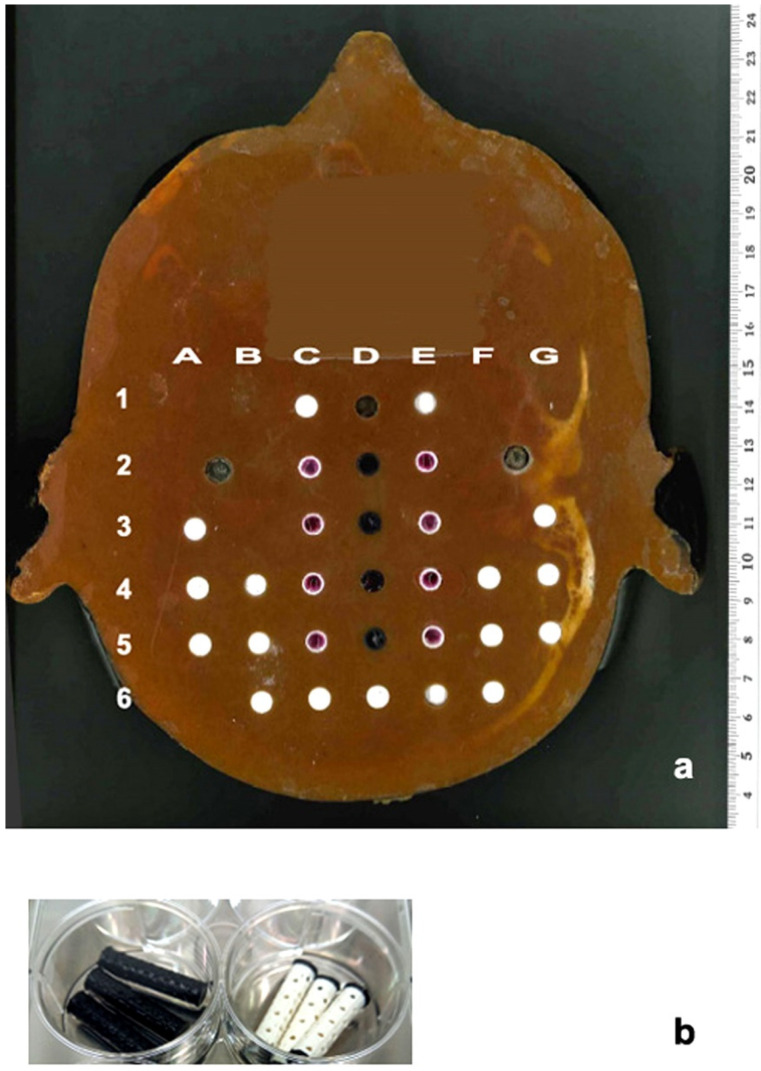
Slice 4 of the Alderson phantom overlayed with a grid which allows the correlation of every single sample with its position during irradiation. 3D cultures are placed into positions C–E2–5 (**a**). 3D printed tubes, produced from biocompatible material as shells for 3D cell cultures in an agar matrix, to be inserted into different positions in the Alderson phantom (**b**).

In the example shown in Figure 8, the positions D2–5 contain the 3D cell culture samples to be irradiated, the positions C2–5 and E2–5 contain samples for the analysis of bystander effects.

## 4. Discussion

In small animal experiments of high dose rate radiotherapy, it has been shown that, at equal nominal target doses, the tumor-destructive effects of high dose rate radiotherapy approaches such as broad beam FLASH and MRT are equal or higher than with conventional radiotherapy techniques at low dose rates. At the same time, the preservation of normal tissue function is significantly higher with high dose rate radiotherapy, compared to conventional radiotherapy. Most of these studies were focused on normal tissue preservation and tumors in the brain [18,19,39,40,41,42] and the lung [12,43,44], which are considered fairly radioresistant in conventional radiotherapy.

The importance to study the effects of high dose rate radiotherapy in veterinary patients before proposing human clinical trials has been recognized because dogs, for instance, suffer from spontaneous tumors which are very similar in size, histology, and natural course of the disease, compared to human patients [45,46]. The first successful veterinary studies were and are conducted with FLASH radiotherapy [21,22] and with MRT, the latter focused on the treatment of dogs with malignant brain tumors [28]. 

The international interdisciplinary efforts to develop high dose rate radiotherapy for clinical trial will profit from the work conducted with the new experimental tool we have developed to extend the research capacity in this field. We defined the key parameters of the incident synchrotron beam at the polychromatic beamline P61A, and designed and constructed a mobile experimental setup which can be inserted into the beamline environment on demand. 

Contrary to conventional radiotherapy, where the gantry is moved around the patient, the beam position is fixed at the synchrotron and the patient, or the sample needs to be moved through the beam. Typically, the dose deposited in the target is controlled by the speed of vertical movement through the beam. While this is the only directional movement required of the sample for unidirectional irradiation, bi-directional or multi-directional irradiation requires extremely exact repositioning or movement of the sample around an isocenter. The limiting factor at P61A, which also sets the beamline apart from dedicated biomedical beamlines, is the width of the incident beam. If the width of the intended target is small enough to be covered by the narrow incident beam, the target dose can be administered during one vertical movement of the sample through the beam. If the target is wider than the incident beam, irradiation fields need to be patched laterally and a second vertical movement through the beam is required after precise lateral translation of the sample.

To satisfy the special technical parameters at beamline P61A, we developed two suitable models for biomedical work at this beamline, both utilizing 3D cell cultures. Contrary to the commonly used 2D cell cultures, 3D cell cultures can be easily modified in size to fit the available beamline parameters and can be presented for irradiation in such a way that contamination of the beamline with biological material is prevented. Two different phantoms, not originally intended for work at the synchrotron, were modified to permit sample presentation at the narrow synchrotron beam of PETRA III. The pattern of energy distribution is very dependent on parameters like beam attenuation and scattering due to material, size, and shape of a phantom. In our first model, we use a phantom representing the size and shape of a mouse, produced in an additive manufacturing process from tissue equivalent materials. Custom-sized pieces of 3D cell cultures generated in a bioprinting process can be inserted into the phantom at key positions along pieces of self-developing Gafchromic™ film into prefabricated slots for irradiation. Thus, changes detected in the biological sample can be directly related to the irradiation-induced changes on the film, evaluating the new biodosimetry method against the well-established film dosimetry. One example for use would be to compare the intensity of gammaH2AX staining after microbeam irradiation [47] with film dosimetry. 

Our second model is based on a commercially available phantom in which 3D cell culture samples are inserted. This phantom is regularly used for clinical dosimetry. Thus, size and absorption parameters are similar to that of human tissue. Two aspects make this model especially appealing for in vitro studies: First, tumor depth and the conditions of scatter in the head can be reliably simulated by fitting the slice loaded with cell culture tubes in its original position in the phantom. Second, in one single irradiation procedure, it is possible to irradiate samples at different depths similar to the situation of clinical radiotherapy. This aspect becomes even more interesting if 3D cultures of tumor samples are surrounded by 3D samples generated from normal, non-malignant cells. At beamline P61A, irradiation can be easily conducted across one row or one column of a phantom slice. To study larger irradiation volumes, a transfer of the experiment to a dedicated biomedical beamline would be desirable. 

## 5. Conclusions

We created a tool for biomedical high dose rate irradiation research at the synchrotron beamline P61A. Despite the narrow beam width typical for PETRA III beamlines, two suitable models for in vitro studies have already been developed which might prove useful even for the work at dedicated biomedical beamlines in the future.

## Data Availability

Original data are available from the first author on request.
